# *Nitrospina* bacteria in a rocky intertidal habitat (Quintay Bay, central Chile)

**DOI:** 10.1002/mbo3.646

**Published:** 2018-05-24

**Authors:** Daniela V. Yepsen, Héctor A. Levipan, Verónica Molina

**Affiliations:** ^1^ Programa de Doctorado en Ciencias con mención en Manejo de Recursos Acuáticos Renovables Universidad de Concepción Concepción Chile; ^2^ Centro de Investigación Marina Quintay (CIMARQ) Facultad de Ecología y Recursos Naturales Universidad Andres Bello Valparaíso Chile; ^3^ Programa de Biodiversidad Departamento de Biología Observatorio de Ecología Microbiana Facultad de Ciencias Naturales y Exactas Universidad de Playa Ancha Valparaíso Chile

**Keywords:** 16S‐rRNA gene transcript, intertidal bacteria, nitrite oxidation, *Nitrospina*, rocky intertidal habitat, stress tolerance

## Abstract

*Nitrospina* bacteria are among the most important nitrite oxidizers in coastal and open‐ocean environments, but the relevance of the genus contrasts with the scarceness of information on their ecophysiology and habitat range. Thus far, *Nitrospina* bacteria have been the only nitrite oxidizers detected at high abundance in Chilean coastal waters. These levels are often higher than at other latitudes. In this study, the abundance of 16S‐rRNA gene transcripts of *Nitrospina* (hereafter just transcripts) was measured by reverse transcription quantitative PCR in a rocky intertidal gradient and compared with the nearshore counterpart off central Chile (~33°S). Rocky pond transcripts were also compared with the taxonomic composition of the macrobiota and bacterioplankton (by 16S‐rRNA gene‐based T‐RFLP) in the intertidal gradient. Transcripts increased from warmer, saltier, and low‐nitrite ponds in the upper intertidal zone (19.5 ± 1.6°C, 39.0 ± 1.0 psu, 0.98 ± 0.17 μmol/L) toward cooler, less salty, and high‐nitrite ponds (17.8 ± 2.6°C, 37.7 ± 0.82 psu, 1.23 ± 0.21 μmol/L) from middle and low zones. These varied from ~1,000 up to 62,800 transcripts. This increasing trend in the number of transcripts toward the lower zone was positively associated with the Shannon's diversity index for the macrobiota (*r* = .81, *p* < .01). Moreover, an important increase in the average number of transcripts was observed in ponds with a greater number of fish in the upper (7,846 transcripts during 2013) and lower zones (62,800 transcripts during 2015). Altogether, intertidal and nearshore transcripts were significantly correlated with nitrite concentrations (*r* = .804, *p* ˂ .01); rocky pond transcripts outnumbered nearshore ones by almost two orders of magnitude. In summary, rocky ponds favored both the presence and activity of *Nitrospina* bacteria that are tolerant to environmental stress. This in turn was positively influenced by the presence of ammonia‐ or urea‐producing macrobiota.

## 1 INTRODUCTION

Canonical nitrification is a two‐step aerobic reaction that has a key role in marine nitrogen cycling (Ward, 2008). The first reaction is the oxidation of ammonia and/or ammonium into nitrite by ammonia‐oxidizing bacteria and archaea. The second reaction is nitrite‐fueled and catalyzed by nitrite‐oxidizing bacteria (NOB) to produce nitrate. Recently, one‐step complete nitrification (comammox *Nitrospira*) has been confirmed in laboratory cultures (Daims et al., 2015; van Kessel et al., 2015). This was also suggested for some yet‐uncultured *Nitrospira*‐like bacteria through metagenomic approaches (Pinto et al., 2016; Wang et al., 2017). However, there is no research offering sound evidence on the presence of comammox genes in marine samples, and most nitrate production in marine environments is thought to be NOB‐driven.

The NOB guild is a functional group that includes phylogenetically distant bacteria belonging to hitherto seven genera distributed over four phyla including the phylum *Nitrospinae* (Daims, Lücker, & Wagner, 2016). Members of this phylum have been detected in the field and are basically related to marine chemolithotrophic nitrification (Pachiadaki et al., 2017). *Nitrospina*‐like bacteria have been detected from the surface down to the deep ocean (DeLong et al., 2006; Nunoura et al., 2015; Santoro, Casciotti, & Francis, 2010; Watson & Waterbury, 1971) including in oxygen minimum zones (OMZs) (Beman, Shih, & Popp, 2013; Fuchsman, Kirkpatrick, Brazelton, Murray, & Staley, 2011; Labrenz, Jost, & Jurgens, 2007; Zaikova et al., 2010), oxic/anoxic sediments (Davis, Stakes, Wheat, & Moyer, 2009; Jorgensen et al., 2012; Rani, Koh, Rhee, Fujitani, & Park, 2017), and deep estuarine environments (Colatriano et al., 2015). These bacteria reach abundances as high as 9.25% of total 16S‐rRNA gene sequences recovered in bacterial sequence libraries obtained from the eastern tropical North Pacific (Beman et al., 2013), and up to one‐third of the bacterial 16S‐rRNA gene sequences in deep hypersaline ponds in the Red Sea (Ngugi, Blom, Stepanauskas, & Stingl, 2016).

A study conducted in the subtropical eastern South Pacific (ESP) off Concepcion (~36°S, central‐southern Chile) showed that *Nitrospina*‐like bacteria were the only known NOB detected by pyrosequencing. They accounted for up to 5% of the total bacteria in OMZ‐influenced waters; they were also detectable through reverse transcription quantitative PCR (RT‐qPCR) in surface waters (Levipan, Molina, & Fernandez, 2014). Two recent studies described sequences affiliated only with *Nitrospina* in connected oceanographic areas off the Chilean coast, namely, the ESP off Concepcion (Bristow et al., 2016) and eastern tropical South Pacific (ETSP) off Iquique (~20° S, northern Chile) (Pachiadaki et al., 2017). Therefore, other ubiquitous NOB in marine ecosystems such as members of genera *Nitrospira* (Hawley, Brewer, Norbeck, Pasa‐Tolic, & Hallam, 2014; Lüke, Speth, Kox, Villanueva, & Jetten, 2016) and *Nitrococcus* (Füssel et al., 2012, 2017) appear to be absent or occur at very low abundance in Chilean waters (Léniz, Murillo, Ramírez‐Flandes, & Ulloa, 2017; Ngugi et al., 2016).

In contrast to ammonia oxidizers, *Nitrospina*‐NOB has received much less attention; hence, there is a general lack of information on their ecophysiological features and habitat amplitude (Daims et al., 2016) outside of coastal and open‐ocean environments. In this context, rocky intertidal systems offer an ideal setting to study the physiological versatility of yet‐uncultured *Nitrospina*, because these environments are characterized by strong physical‐chemical gradients (temperature, salinity, desiccation) and complex biotic interactions among the organisms living there (e.g., competence, predation, commensalism) (Bertness, Leonard, Levine, Schmidt, & Ingraham, 1999; Bruno, Stachowicz, & Bertness, 2003; Hay et al., 2004). Herein, the 16S‐rRNA gene‐based transcriptional activity of *Nitrospina* was studied in a rocky intertidal habitat and compared with its nearshore counterpart and environmental changes in the upper, middle, and low intertidal zones as well as with intertidal biotic conditions.

## 2 MATERIALS AND METHODS

### 2.1 Study site and physical‐chemical characterization

#### 2.1.1 Rocky intertidal gradient and sampling

**Figure 1:**
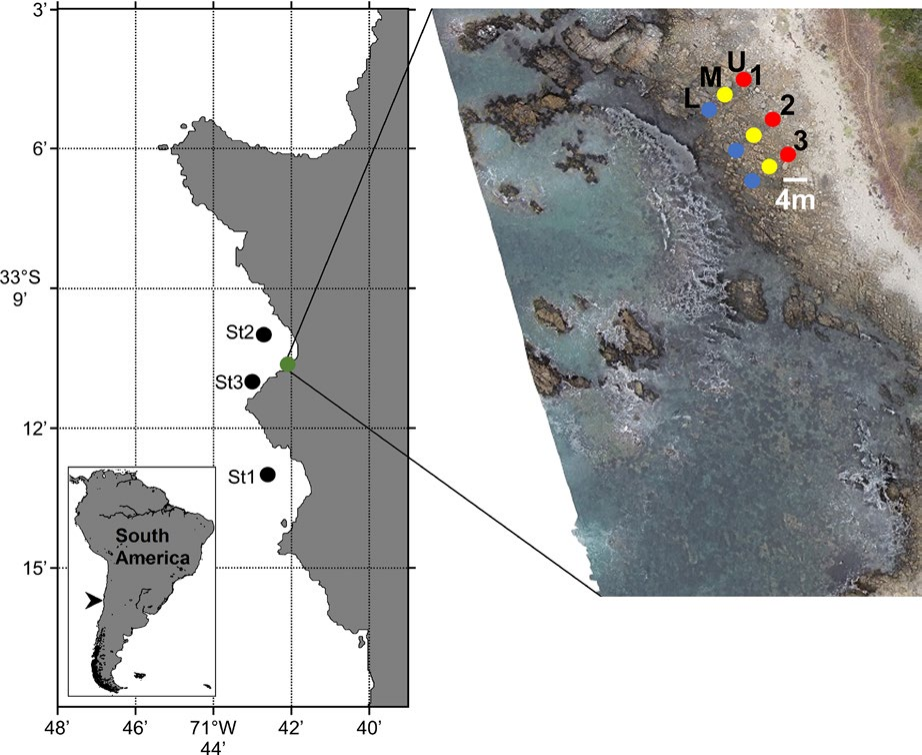
Study site in central Chile (~33° lat. S). Green and black dots denote the rocky intertidal habitat (“El Litre” beach in Quintay Bay) and nearshore marine stations, respectively. An aerial picture viewed from a drone shows triplicate rocky ponds at the low (L), middle (M), and upper (U) intertidal zones

A rocky intertidal habitat in “El Litre” Beach of the Quintay Bay in central Chile (33°11′22″ S ‐ 71°41′39″ W) was visited twice at midday in autumn of 2013 (April) and summer of 2015 (January). Samples were collected from three rocky ponds (~1‐m^2^ area and ≤50 cm depth) within three zones across an intertidal gradient: low (ponds L1 to L3), middle (ponds M1 to M3), and upper zones (ponds U1 to U3) (Figure 1). The intertidal zones were chosen based on their exposure to air between high and low tides (Stephenson & Stephenson, 1949) so that the low zone was exposed to air at lower tides. The upper zone was submerged only at higher tides, and the middle zone was characterized by an intermediate period of exposure to air and interruptions in the distribution of *Ulva* sp. and other algae (Table 1). The salinity was measured in each pond using a salinity refractometer S/Mill‐E (Atago Co. Ltd, Tokyo, Japan) with a range of zero to 100.0 psu (graduation 1.0). The temperature and dissolved oxygen were measured using a handheld meter Oxi 330i (WTW GmbH, Weilheim & Co. KG., Germany) equipped with a CellOx 325 DO probe (range of 0–19.99 mg O_2_/L and resolution at 0.01) and integrated temperature sensor (range of 0‐50.0°C with 0.1°C resolution).

**Table 1:** Relative abundance (% mean ± *SD*) of organisms that structured the intertidal macrobiota in the autumn of 2013

Organism	Intertidal zone		
	Upper	Middle	Low
*Fissurella* sp.	0.64 ± 1.11	1.01 ± 0.96	9.65 ± 8.44
*Perumytilus purpuratus*	29.15 ± 12.78	18.23 ± 18.82	4.44 ± 7.70
*Echinolittorina peruviana*	53.53 ± 11.06	33.63 ± 31.83	19.66 ± 25.88
*Chiton* sp.	0	3.03 ± 4.61	5.35 ± 4.83
*Acanthopleura granulata*	0	0	5.42 ± 5.05
*Betaeus emarginatus*	5.66 ± 6.13	1.72 ± 1.49	6.46 ± 5.60
*Petrolisthes violaceus *	1.68 ± 1.23	3.20 ± 3.51	8.82 ± 2.23
*Homalaspis plana*	0.14 ± 0.25	1.05 ± 1.83	3.87 ± 2.16
Unidentified crab	0	0	2.22 ± 3.85
*Balanus* sp.	0	5.39 ± 9.34	0
*Helcogrammoides* sp.	0	11.11 ± 19.24	12.06 ± 11.11
*Girella laevifrons*	9.20 ± 15.93	0	0
*Heliaster helianthus*	0	21.63 ± 31.83	22.05 ± 4.67
*Corallina* sp.^a^	0	11.67 ± 16.07	21.67 ± 14.43
*Ulva* sp.^a^	0	61.67 ± 32.53	50.00 ± 10.00
*Colpomenia* sp.^a^	0	16.67 ± 10.41	6.67 ± 5.77
*Codium* sp.^a^	0	0	8.33 ± 14.43

a Coverage percent. This biotic variable corresponds to the rocky area covered with a specific alga. This was estimated from the number of sub‐quadrants within a 0.25‐m^2^ quadrant that were occupied by an individual alga and expressed as percentage of total sub‐quadrants.

Duplicate seawater samples (100 ml) for nitrite determinations were collected and filtered using a GFF filtration unit in the field and stored in sterile 125 ml Nalgene bottles inside cooler containers with frozen gel packs during transport to the laboratory (~2 hr in travel distance). They were then stored at −20°C for 3 days until analysis. Seawater samples were thawed and analyzed spectrophotometrically using standard colorimetric analysis (detection limit ~0.01 μmol/L) following the procedure described by Strickland and Parsons (1972). Environmental data were available only for the 2013 sampling.

#### 2.1.2 Nearshore stations and sampling

Seawater samples were collected at different depths (0, 10, 20, and 40 m) from three shallow coastal stations off “El Litre” Beach in the summer of 2011 (December): St.1 (33°12.9996′ S‐ 71°42.6′ W), St.2 (33°10.0002′ S ‐ 71°42.6996′ W), and St.3 (33°10.99998′ S ‐ 71°42.9996′ W) (Figure 1). The salinity, temperature, and oxygen dissolved were measured using a CTD instrument equipped with an oxygen sensor (RBR Ltd., Ottawa, Canada).

#### 2.1.3 Microbial and macrobiota samplings

Triplicate seawater samples (100 ml) were collected from coastal and intertidal sites and then concentrated by filtration on GVWP filters (pore size 0.22 μm, MF‐Millipore) using disposable syringes (60 ml) and sterile swinnex filter holders (48 mm). Microbe‐loaded filters were soaked with 300 μl of RNA later (Thermo Fisher Scientific, Carlsbad, CA, USA) in cryovials, transported to the laboratory in coolers, and then stored at −20°C until further processing for RNA extraction.

The macrofauna (≥1 cm) of Mollusca, Echinodermata, Crustacea, and Perciformes was quantified in rocky ponds only in the fall of 2013 except for perciforms (quantified in the two intertidal samplings). The coverage percent of algae (*Codium* sp., *Colpomenia* sp., *Corallina* sp., and *Ulva* sp.) was measured using 0.25 m^2^ quadrants divided into 25 equivalent sub‐quadrants.

### 2.2 Extraction of total RNA

The RNA was extracted from microbe‐loaded filters using the Ambion RNA extraction kit (Thermo Fisher Scientific) according to the manufacturer's suggestions with some modifications. Briefly, a mechanical cell disruption step was performed using 200 μm‐diameter zirconium beads (OPS Diagnostics, Lebanon, NJ, USA) and a mini bead‐beater (Mini‐Beadbeater‐8, BioSpec Products Inc., Bartlesville, OK, USA). This device was programmed with two agitations at ~3,000 strokes/min for 30 s each and pauses of 30 s between agitations. The concentrations and A_260_/A_280_ ratios (quality) of RNA extracts were determined on a Synergy MxMicroplate Reader (BioTek Instruments, Winooski, VT, USA). Traces of contaminant DNA in the RNA preparations were removed with the TURBO DNA‐free kit (Thermo Fisher Scientific).

### 2.3 Two‐step RT‐qPCR assay for *Nitrospina* bacteria

DNase‐treated RNA samples were used to synthetize cDNAs with the ImProm‐II Reverse Transcription System (Promega, Madison, WI, USA) and reverse primer NitSSU_286R (5′‐CCYCTCAGGCCGGCTA‐3′; Levipan et al., 2014). The resulting cDNAs were used as templates for qPCR experiments targeting *Nitrospina* 16S‐rRNA genes using the primer combination NitSSU_130F (5′‐GGGTGAGTAWCACGTGAATAA‐3′; Levipan et al., 2014) and NitSSU_286R. This primer set is specific for *Nitrospina*‐like bacteria and was modified from the primer pair designed by Mincer et al. (2007). *Nitrospina* 16S‐rRNA gene amplicons were quantified from triplicate samples following procedures previously described (Levipan et al., 2014) regarding the chemistry of qPCR reactions, amplification program, and device used. For quantification, the clone ST180811‐50 m11 (access number KF452035) was used to prepare a 6‐point standard curve that started from 1 × 10^7^ gene copies using 10‐fold dilutions (efficiency and *r* ^2^ values of 83.7% and .99, respectively). The copy number of the respective clone was calculated by dividing its DNA concentration (in ng/μl) by its mass (in ng) calculated using the following formula: mass = [(n) × (M/N_A_)] × 10^9^ where n is the clone size (vector plus insert, in bp); M, is the average molecular weight of a base pair (660 g/mol); N_A_, is the Avogadro's number (60,221 × 10^23^ bp/mol); and 10^9^ is the factor to convert grams to nanograms. *Nitrospina* amplicons with the expected size were verified by analyzing melting curves and visualized through standard agarose gel electrophoresis.

### 2.4 Terminal restriction fragment length polymorphism (T‐RFLP) analysis of active bacterial communities

The T‐RFLP analysis of 16S‐rRNA gene amplicons was performed using universal bacterial primers 5‐carboxyfluorescein (NED and FAM)‐8F (5′‐AGAGTTTGATCCTGGCTCAG‐3′) and 519R (5′‐GWATTACCGCGGCKGCTG‐3′) with the intertidal RNA samples collected in 2013. We used the procedures described by Morán et al. (2008). Briefly, cDNA templates (10 ng) were mixed with puReTaq Ready‐To‐Go Polymerase Chain Reaction Beads (GE Healthcare, Buckinghamshire, UK) following the manufacturer's instructions. The PCR program included denaturation at 95°C for 5 min followed by 35 amplification cycles. Every cycle consisted of denaturation at 95°C for 45 s, primer annealing at 56°C for 45 s, and a 45‐s primer extension at 72°C. The program ended with a final extension at 72°C for 7 min. The PCR products were quantified and then digested overnight with *Msp*I and *Hha*I restriction enzymes at 37°C per the manufacturer's instructions. The digestion was stopped by cold precipitation using sodium acetate (3 mol/L) and ethanol (99.5%) at −80°C for at least 1 hr followed by centrifugation at 16,060 g rpm for 30 min. The resulting pellet was washed using cold ethanol (70%), centrifugated again under similar conditions, dried in a laminar‐flow chamber to avoid contamination, and resuspended in 30 μl of PCR grade water with thorough mixing. These samples were quantified on a Qubit fluorometer using the instructions from the manufacturer (Thermo Fisher Scientific). The samples and internal standard (GSLIZ 500 fragment ladder) were run on an ABI 3130 automated sequencer (Applied Biosystems, Foster City, CA) at the Pontificia Universidad Católica of Chile (PUC).

The T‐RFLP fragment peak (50–500 bp) data were extracted into excel format with the Peak Scanner Software. We only describe results generated with the *Msp*I enzyme because the two restriction enzymes showed the same pattern.

### 2.5 Data analysis

The 16S‐rRNA‐based transcriptional activity of *Nitrospina* was expressed as number of 16S‐rRNA gene transcripts per nanogram of total RNA in seawater samples. Their relationship with abiotic (NO_2_^−^, salinity, dissolved oxygen, and temperature) and biotic variables (macrobiota and bacterial operational taxonomic units, OTUs) was determined by the Spearman (rs) and Pearson correlations. The richness and diversity indices were calculated with the PAST software version 3.04 (Hammer, Harper, & Ryan, 2001). In addition, a nonparametric Mann–Whitney test among *Nitrospina* transcripts and fish abundances was performed using PAST with 2013 and 2015 data. Significant differences of physical‐chemical data between intertidal zones were computed by using a one‐way‐ANOVA followed by the Tukey's HSD post‐hoc test (*p < .05).

Fluorescent T‐RFLP peaks were assigned to specific OTUs and expressed as a percentage of an individual OTU to the total fluorescence in each sample (i.e., as bacterial abundance percentage). The cluster analysis of square‐root‐transformed OTU data was performed using the Jaccard's proximity coefficient and UPGMA clustering algorithm, with a bootstrap value set of 1,000 times. The Jaccard's coefficient was equal to zero when there were no species or OTUs to share among the rocky ponds. The value was 1 when the ponds shared identical compositions in species or OTUs.

## 3 RESULTS

### 3.1 Physical‐chemical conditions in rocky intertidal ponds and nearshore stations

**Figure 2:**
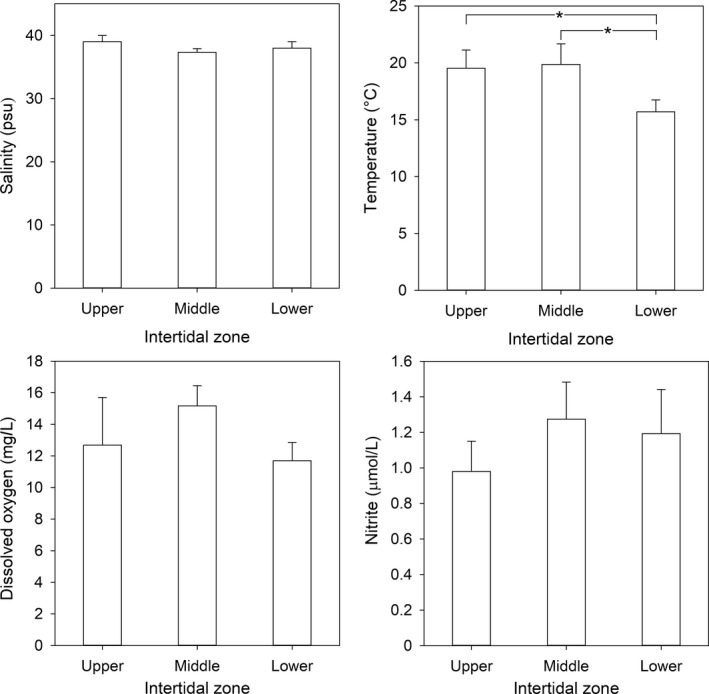
Environmental variables along the intertidal gradient during the fall of 2013. Variations in duplicate measurements for salinity, temperature, and oxygen from a same rocky pond were not detected. Data are average values (± standard deviation) of measurements performed at three ponds per zone. Statistically significant differences between intertidal zones are denoted by an asterisk (*p* < .05)

**Figure 3:**
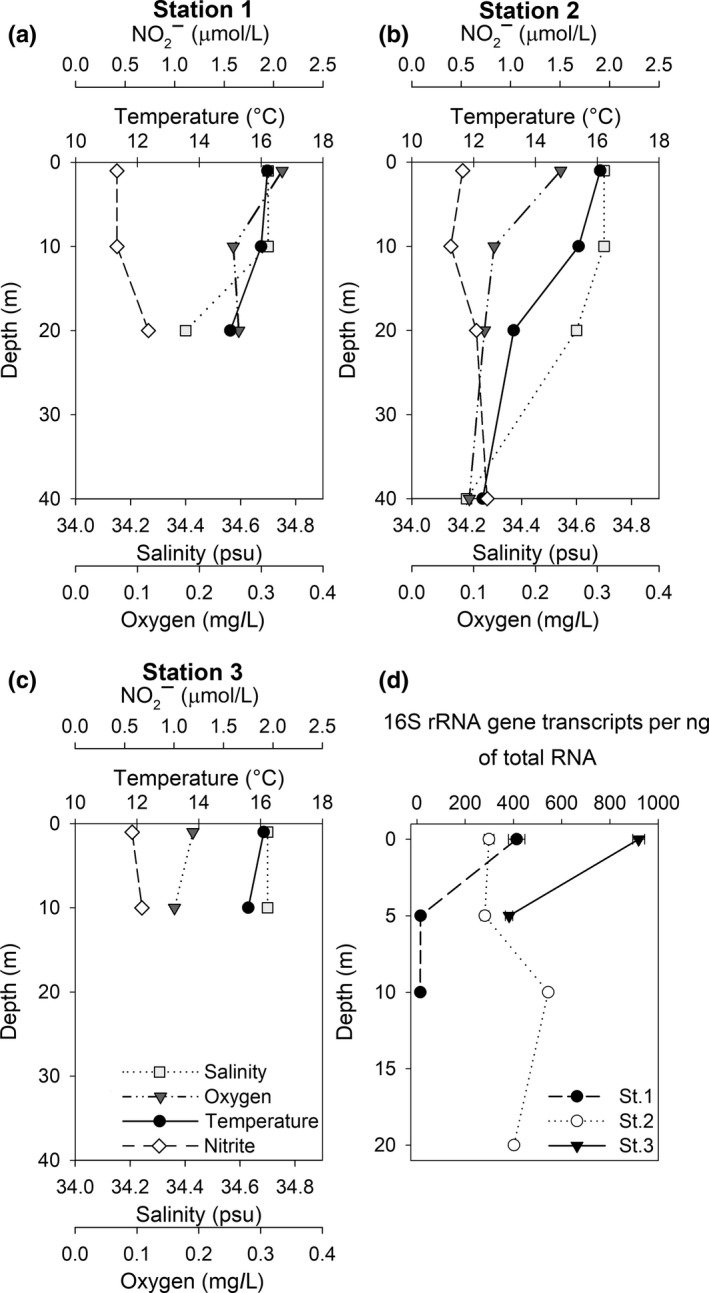
Physical‐chemical variability and 16S‐rRNA‐based transcriptional activity of Nitrospina at three nearshore marine stations. Salinity, dissolved oxygen, temperature, and nitrite at the (a) station 1, (b) station 2, and (c) station 3. Physical‐chemical variables and (d) Nitrospina activities are mean values ± standard deviation (n = 3)

Salinity, temperature, dissolved oxygen, and nitrite concentration were determined in the upper, middle, and low intertidal zones in the fall of 2013 (Figure 2) as well as in the coastal adjacent area (Figure 3). The upper, middle, and low intertidal zones showed average temperatures of 19.5 ± 1.6°C, 19.9 ± 1.8°C, and 15.7 ± 1.0°C, respectively. The most conservative variable was the salinity, which varied from 39 ± 1.0 psu in the upper zone to 37.7 ± 0.82 psu in intermediate and lower ponds. In general, dissolved oxygen concentrations were higher in rocky ponds from the middle zone (15.2 ± 1.3 mg O_2_/L), while the lowest value (10.4 mg O_2_/L) was measured in the pond U1 from the upper zone (Figure 2). Nitrite concentrations ranged from 0.98 ± 0.17 μmol/L in the upper zone to 1.27 ± 0.21 μmol/L in the middle zone (Figure 2).

Coastal seawater temperatures varied between 12.3 and 16.2°C. The thermocline in the deepest coastal station was between 10 and 20 m depth (St2.; Figure 3b). The salinity in coastal stations varied between 34.2 and 34.7 psu (Figure 3a, b, and c). Dissolved oxygen concentrations ranged from 0.09 to 0.33 mg/L in the water column of the three coastal stations (Figure 3a, b, and c), while nitrite concentrations did not exceed the 0.76 μmol/L threshold and showed a slight increase with depth (Figure 3a, b, and c).

### 3.2 Macrobiota composition and bacterial community structure in the intertidal habitat

The macrobiota composition in the intertidal gradient was dominated by *Perumytilus purpuratus* and *Echinolittorina peruviana* in the middle and upper rocky ponds (Table 1). In addition, fish *Girella laevifrons* made up an important fraction (27%) of total macrobiota in the pond U3 (Table 1). Diversity indices of species richness (S) and Shannon's diversity (H) for the macrobiota were higher in low ponds (S = 13, H = 2.39) than upper ponds (S = 4, H = 1.26) (Table 2). There was a significant relationship between the H‐indices and nitrite concentrations (*r* = .738, *p* < .01). In addition, the abundance of *Chiton sp*. and *P. purpuratus* was significantly correlated with the nitrite concentration (*r* = .79, *p* < .01) and temperature (*r* = .71, *p* < .01) in the intertidal habitat, respectively.

**Table 2:** Diversity indices of species richness and Shannon's diversity for bacterial community and macrobiota in the intertidal habitat during the fall of 2013

		Upper zone (U)	Middle zone (M)	Low zone (L)
Bacterial community	Richness	15–17	26	19–23
	Shannon	2.153–2.248	2.555–2.702	2.267–2.565
Macrobiota community	Richness	4–5	6–12	8–13
	Shannon	1.259–1.384	1.62–2.16	1.97–2.389

**Figure 4:**
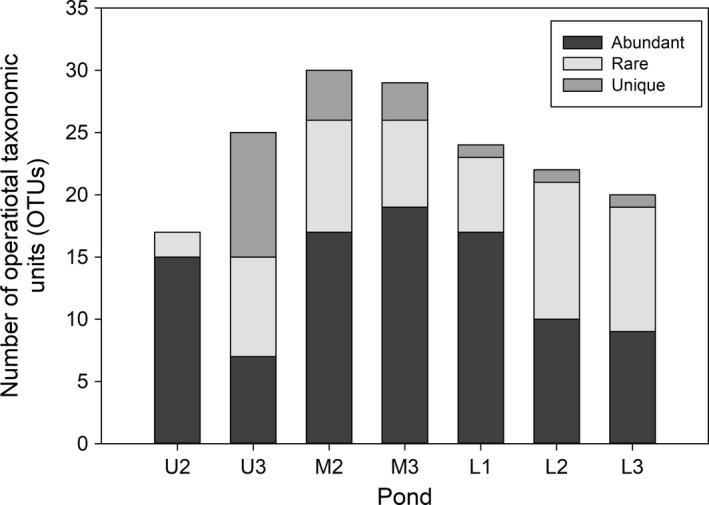
Total number of abundant, rare, and unique OTUs into rocky ponds found in different intertidal zones (U  =  Upper, M  =  middle, and L  =  low) during the fall of 2013. Rare and abundant OTUs were defined as OTUs with abundances of < and ≥ 1%, respectively. Samples from ponds U1 and M1 were lost during the procedure

The bacterial community was made up of 56 OTUs with variable abundance and distribution in the intertidal gradient during the fall of 2013. The upper and middle rocky ponds showed a higher number of unique OTUs (Figure 4), for example, pond U3 harbored 10 exclusive OTUs of a total number of 25 OTUs. On the other hand, abundant OTUs (i.e., with relative abundances ≥1%) varied between 7 and 15 in the upper zone, 17 and 19 in the middle zone, and from 9 to 17 in the low zone. Low‐frequency or rare OTUs (i.e., with relative abundances <1%) reached higher counts in the low zone (Figure 4). In addition, low‐frequency OTUs included ribotypes (e.g., OTU 77 and OTU 95) that changed their contribution to bacterial communities along the intertidal gradient. These were rare in the upper zone but abundant in low and middle zones. Community structure changes based on the clustering analysis indicated a higher similarity between bacterial communities from low and middle zones compared with the upper zone (Figure S1). The alpha diversity parameters of bacterial communities indicated less diversity in the upper and low zones compared with the middle intertidal zone (Table 2). Changes in the alpha diversity of the bacterial community were not significantly correlated with abiotic or biotic parameters, but they did show a positive association with changes in alpha diversity of the macrobiota (Table 2).

### 3.3 *Nitrospina* 16S‐rRNA gene transcripts in rocky intertidal ponds and nearshore stations

**Figure 5:**
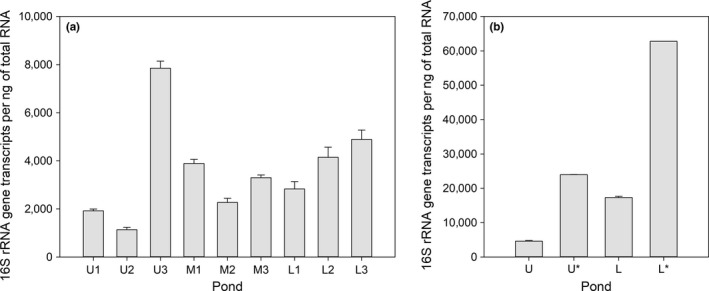
*Nitrospina* transcripts in rocky ponds from different intertidal zones (U  =  Upper, M  =  middle, and L  =  low) during the (a) fall of 2013 and (b) summer of 2015. Ponds in high and low zones with (*) and without fish are compared in (b). The gene transcripts are means ± standard deviations (*n* = 3)

*Nitrospina* transcripts were detected in all intertidal ponds from the 2013 and 2015 samplings. These tended to increase towards the lower portions of the intertidal habitat (Figure 5). One exception to this trend was in pond U3 (upper zone). It showed an average value of 7,846 16S‐rRNA gene transcripts per ng of total RNA (Figure 5a). In general, a higher number of *Nitrospina* transcripts (Figure 5b) was associated with the presence of fish in rocky ponds—this is based on significant differences between ponds with and without fish during 2015 (Mann–Whitney test; *p *˂ .001). Moreover, *Nitrospina* transcripts were positively correlated with H‐indices of macrobiota communities along the intertidal gradient (*r* = .81, *p* < .01).

**Figure 6:**
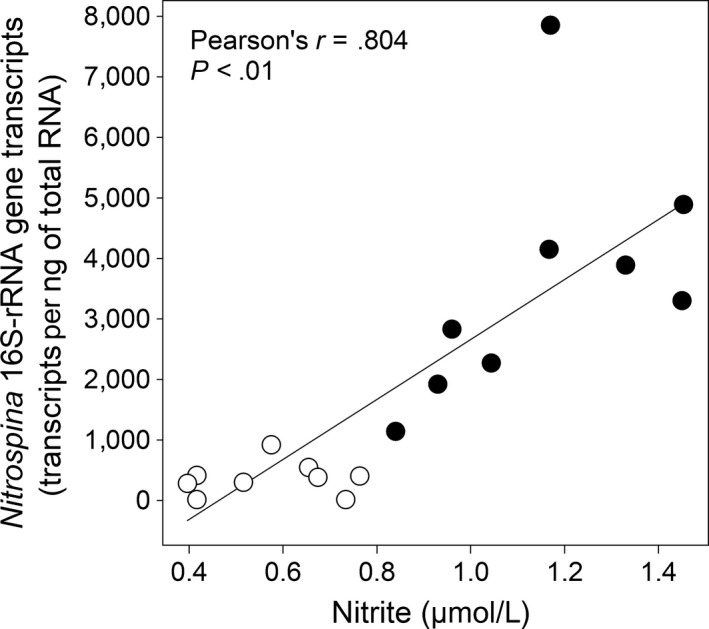
Relationship between nitrite concentrations and *Nitrospina* transcripts. Open and filled circles indicate seawater samples collected from nearshore and intertidal habitats, respectively. Intertidal seawater samples correspond to those collected during 2013

The 16S‐rRNA‐based transcriptional activity of *Nitrospina* in surface waters from the two shallowest and nearshore stations was equal to 414 (St.1) and 919 (St.3) transcripts per ng of total RNA (Figure 3d). *Nitrospina* transcripts decreased with increasing depth at shallow stations; we noted the opposite trend at the deeper nearshore station St.2 (Figure 3d). Altogether, *Nitrospina* transcripts from intertidal and nearshore seawaters showed a positive correlation with nitrite concentrations (*r* = .804, *p* < .01) (Figure 6).

## 4 DISCUSSION

Studies on microbial ecology in rocky intertidal systems are rare—particularly along the Chilean coastline. This study highlights the presence and high 16S‐rRNA‐based transcriptional activity of *Nitrospina* bacteria as potential key players in nitrogen cycling of a rocky intertidal habitat off central Chile (~33° lat. S). We detected an intertidal‐stimulated transcriptional activity in *Nitrospina* that reached up to 62,800 transcripts per ng of total RNA (Figure 5b). This number of transcripts is almost two orders of magnitude higher than the average values detected in nearshore stations (Figure 3d) including other areas off the Chilean coast. The number of intertidal *Nitrospina* transcripts exceeded the transcript range reported for seawater samples collected in the first 20 m depth off central‐southern Chile (~36° S). It was only comparable with values found in the OMZ‐influenced layer (at ~80 m depth) using similar methodological approaches (Levipan et al., 2014, 2016).

In general, the low and middle zones showed greater number of *Nitrospina* transcripts than the upper zone, and these co‐occurring with changes in biotic and abiotic variables. Thus, the *Nitrospina* transcripts were positively associated with macrobiota's H‐indices (*r* = 0.81, *p* < .01) and nitrite concentrations (Figure 6). One exception to this trend was detected in the pond U3 (upper zone) during 2013. This showed the greatest number of *Nitrospina* transcripts in co‐occurrence with both a differential structure of the bacterial community (Figure S1) and conspicuous presence of *G*. *laevifrons* (Table 1). A second sampling was performed during the summer of 2015, and this suggested that the transcriptional activity of *Nitrospina* increases in ponds with fish compared to ponds where fish were at low abundance or absent independent of the zone along the intertidal gradient (Mann–Whitney test; *p* ˂ .001, Figure 4b).

Two mechanisms could explain a potentially fish‐stimulated response in the number of *Nitrospina* transcripts. The ammonia excretion from fish can favor the first reaction of nitrification (Eikebrokk & Piedrahita, 1997). This in turn fuels the *Nitrospina*‐catalyzed nitrite oxidation. Thus, the increase in intertidal *Nitrospina* transcripts may be related to changes in ammonia oxidation rates similar to *Nitrospina* abundance in other coastal areas (Luria, Amaral‐Zettler, Ducklow, & Rich, 2016). Alternatively, urease‐like genes in *Nitrospina* (Ngugi et al., 2016) could convert fish‐derived urea into ammonium. This ultimately will lead to favored coupling between *Nitrospina* and ammonia oxidizers (Pachiadaki et al., 2017). There is experimental evidence that fish metabolism stimulates the nitrification including the transcriptional activity of *Nitrospina* (Elizondo‐Patrone, Hernández, Yannicelli, Olsen, & Molina, 2015). Here, a positive association was found between *Nitrospina* transcripts and nitrite concentrations in intertidal and nearshore seawaters (Figure 6). This suggests the crucial role of *Nitrospina* in the nitrate pool dynamics depending on catalytic substrate offers. In fact, there is a recent report on nitrite as the major environmental forcing in modulating the ecological niche of putative *Nitrospina*‐like NOB in the ETNP (Sun, Ji, Jayakumar, & Ward, 2017).

Intertidal areas with strong physical‐chemical gradients likely favor versatile microbial groups such as *Nitrospina*. The wet‐dry alternation during tidal cycles might enhance the *Nitrospin*a's activity and generate a rapid protein turnover triggered by stress conditions in the intertidal habitat versus their nearshore counterparts. Significant correlations were found between *Nitrospina* transcripts in intertidal‐coastal seawaters and potential stressors as salinity (Pearson's *r* = .74, *p* < .01) and dissolved oxygen (Pearson's *r* = .69, *p* < .01). Interestingly, the primer set used here detects two single‐cell‐amplified genomes (SAGs) that were studied by Ngugi et al. (2016) in a brine‐seawater interface (SCGC AAA799‐A02 and SCGC AAA799‐C22). These were designated as “*Candidatus* Nitromaritima” (or clade 1) and show a genomic potential to couple nitrite oxidation at salinities as high as 11.2% (~112 psu). In fact, closely related sequences to *Ca*. Nitromaritima were numerically dominant in OMZ metagenomes in the ETSP off northern Chile as well as in metatranscriptomes in the subtropical ESP off Concepción (Léniz et al., 2017).

The above‐mentioned findings and our results suggest that a large fraction of coastal *Nitrospina* bacteria can thrive at salinities close or slightly above 4% without drawbacks. This threshold is 40 g NaCl/L and is the optimum salinity reported for the most halophilic nitrifying strain (Koops, Böttcher, Möller, Pommerening‐Röser, & Stehr, 1990). *Nitrospina* bacteria may tolerate wide fluctuations in oxygen concentrations that could vary from anoxia (Garcia‐Robledo et al., 2017) to well‐oxygenated conditions as reported here. Moreover, *Nitrospina* bacteria are resilient to solar radiation (Levipan et al., 2016), which is an important environmental driver in the upper intertidal zone. However, this was not measured here. Therefore, more ecological and biochemical insights into yet‐uncultured members of the genus *Nitrospina* are needed, including techniques for generating axenic cultures to unveil their physiological and biogeochemical potential. We propose that rocky intertidal habitats are ideal ecosystems to explore *Nitrospina* ecophysiology because these areas are directly subjected to multiple climate change parameters. There are also practical logistical reasons to collect data here, for example, access, manipulability, and low‐cost for sampling.

Our study expands the habitat range of *Nitrospina* outside of coastal and open‐ocean environments by including rocky intertidal habitats. Rocky ponds favored both the presence and activity of *Nitrospina* bacteria that were tolerant to environmental stress. This fact suggests that these bacteria could respond positively to the presence of ammonia‐ or urea‐producing macrobiota (particularly fish) at a transcriptional level.

## CONFLICT OF INTEREST

The authors declare no conflict of interest.
